# Single versus two implant-supported mandibular overdentures: a systematic review and meta-analysis of implant survival and prosthetic complications

**DOI:** 10.1186/s40729-025-00647-1

**Published:** 2025-09-26

**Authors:** Ryo Koyama, Hiroshi Shiratsuchi, Akira Hasuike, Tetsuo Ohyama, Takaaki Tamagawa, Akihiko Furukawa, Shunsuke Namaki, Kazumichi Yonenaga

**Affiliations:** 1 Department of Oral and Maxillofacial Surgery II, Nihon University School of Dentistry, 1-8-13 Kanda-Surugadai, Chiyoda-ku, Tokyo, 101-8310 Japan; 2 Department of Implant dentistry, Dental Hospital, Nihon University School of Dentistry, 1-8-13 Kanda-Surugadai, Chiyoda-ku, Tokyo, 101-8310 Japan; 3 Department of Periodontology, Nihon University School of Dentistry, 1-8- 13 Kanda-Surugadai, Chiyoda-ku, Tokyo, 101-8310 Japan; 4 Department of Partial Denture Prosthodontics, Nihon University School of Dentistry, 1-8-13 Kanda-Surugadai, Chiyoda-ku, Tokyo, 101-8310 Japan; 5 Department of Dysphagia Rehabilitation, Nihon University School of Dentistry, 1-8-13 Kanda-Surugadai, Chiyoda-ku, Tokyo, 101-8310 Japan

**Keywords:** Single implant-supported mandibular overdenture, Two implant-supported mandibular overdenture, Prosthetic complication, Implant overdenture, Denture fracture, Mandibular edentulism

## Abstract

**Purpose:**

This systematic review evaluated the implant survival rate and prosthetic complications of single implant-supported mandibular overdentures (1-IOD) and compared them with those of traditionally recommended two implant-supported mandibular overdentures (2-IOD).

**Methods:**

The Preferred Reporting Items for Systematic Reviews and Meta-Analyses (PRISMA) guidelines were used as a reference for reporting this systematic review. The study protocol was prospectively registered in the PROSPERO database (registration number: CRD420250644169). This review included 17 randomized controlled trials that compared 1-IOD and 2-IOD, with a follow-up period of at least 12 months after denture placement. The assessed outcomes included implant survival rate, denture fractures, denture relining, O-ring replacement, and metal housing reattachment. The risk of bias in the included studies was assessed using the Cochrane Risk-of-Bias 2 tool.

**Results:**

This meta-analysis revealed no difference in implant survival rates between the 1- and 2-IOD groups over a 5-year period. In the subgroup analysis, overdenture fractures, denture remakes, and metal housing reattachments were more frequently observed in the 1-IOD group, whereas no statistically significant differences were found in the need for relining or O-ring replacement.

**Conclusions:**

Although 1-IOD may be a less invasive alternative to 2-IOD, careful consideration is necessary because of its increased incidence of prosthetic complications.

## Graphical Abstract

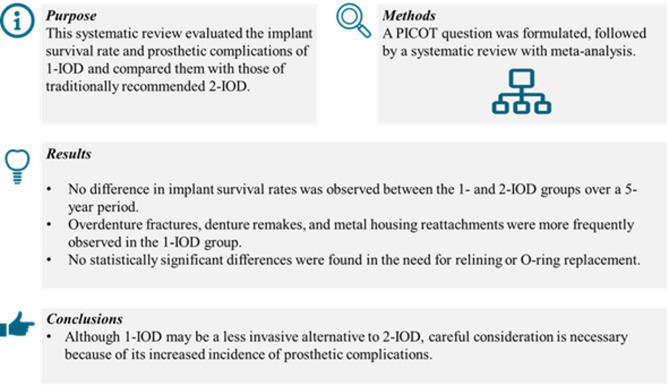

## Background

The restoration of occlusion using complete dentures has long been the most common treatment modality for patients with edentulism. However, conventional complete dentures often significantly reduce masticatory efficiency, particularly in the mandible, where issues such as poor denture stability and insufficient retention pose major clinical challenges [1].

Implant-supported overdentures (IODs) for patients with mandibular edentulism have been reported to offer superior stability and retention compared with conventional removable dentures, thereby improving masticatory efficiency, occlusal force, and patient satisfaction [2–6]. The McGill Consensus Statement in 2002 and the York Consensus Statement in 2009 recommended the use of two IODs (2-IODs) as the first-choice treatment for edentulous mandibles [7, 8]. These statements have contributed to the widespread adoption of 2-IODs as the standard treatment for mandibular IOD. However, the cost of 2-IODs has been reported to be approximately 1.56 to 2.4 times higher than that of conventional complete dentures, which constitutes a significant financial burden for patients [9, 10].

Recently, single IODs (1-IODs) have garnered attention as potential alternatives to conventional complete dentures. Several studies have reported that 1-IOD can enhance masticatory performance compared with conventional dentures, including those by Pinheiro and Kashyap [11, 12]. A systematic review by Nogueira et al. demonstrated that the use of 1-IOD in patients with edentulism significantly improves their quality of life and satisfaction [13]. Additionally, AlSourori et al. reported that the placement of a single implant in the mandibular midline improves denture retention [14].

If 1-IODs can serve as a viable alternative to conventional complete dentures, it may offer advantages over 2-IODs in terms of reduced invasiveness and lower treatment costs, potentially providing significant benefits to patients.

Therefore, this systematic review aimed to evaluate and compare the implant survival and prosthetic complication rates of 1- and 2-IODs based on the existing literature.

## Methods

### Focused question

The “Preferred Reporting Items for Systematic Reviews and Meta-Analyses” (PRISMA) guidelines were used as a reference for reporting this systematic review. The study protocol was prospectively registered in the PROSPERO database (registration number: CRD420250644169). The research questions were framed in the population, intervention, comparison, outcome, and time (PICOT) format:

[P] Patients with complete edentulism wearing 1- or 2-IODs.

[I] 1-IOD.

[C] 2-IOD.

[O] Implant and prosthesis failures.

[T] Timeframe: follow-up period > 12 months.

### Eligibility criteria

Only randomized controlled trials (RCTs) that compared 1- and 2-IODs in accordance with the PICOT format were included.

### Search strategy

A broad systematic literature search was conducted in PubMed, Google Scholar, CENTRAL, and Web of Science, focusing on clinical studies of patients with edentulism treated with 1- or 2-IODs. The final literature search was performed in April 2025.

The PubMed search strategy included a combination of Medical Subject Heading terms, and search expressions were constructed using Boolean operators (OR and AND). To minimize bias and ensure methodological rigor, a qualified librarian developed the search strategy.

The search was conducted using the following key terms: (A) dental implant, (B) denture, (C) mandible, (D) single or double, and (E) randomized.

The PubMed search strategy is presented in Table 1. Similar searches were conducted using Google Scholar, CENTRAL, and Web of Science.

**Table 1:** Database search strategies

A	#1	Dental Prosthesis, Implant-Supported[MeSH Major Topic]
	#2	Dental Implants[MeSH Major Topic]
	#3	Dental Implantation[MeSH Major Topic]
	#4	implant[Title] OR implants[Title]
	#5	#1 OR #2 OR #3 OR #4
B	#6	Denture, Overlay[MeSH Terms]
	#7	overdentur*[Title/Abstract]
	#8	denture*[Title/Abstract] AND over*[Title/Abstract]
	#9	#6 OR #7 OR #8
C	#10	Mandible[MeSH Terms]
	#11	mandib*[Title/Abstract]
	#13	#10 OR #11 OR #12
D	#14	(single[Title/Abstract] OR one[Title/Abstract] OR 1[Title]) AND (implant[Title] OR implants[Title])
	#15	(double[Title/Abstract] OR two[Title/Abstract] OR 2[Title]) AND (implant[Title] OR implants[Title])
	#16	#14 AND #15
E	#17	#5 AND #9 AND #13 AND #16
	#18	“Randomized Controlled Trial” [Publication Type] OR random*[Title/Abstract]
	#19	#17 AND #18

### Data extraction and subgroup analysis

Two reviewers independently and in duplicate screened the titles and abstracts of the studies identified through the literature search. Full-text versions of studies that potentially met the eligibility criteria—or for which the title and abstract provided insufficient information to determine eligibility—were obtained. Any article deemed potentially relevant by at least one reviewer was subjected to full-text screening. These full-text articles were evaluated independently and in duplicate by the same reviewers. Discrepancies were resolved through open discussions; if a consensus could not be reached, a third reviewer was consulted.

Articles that did not meet the eligibility criteria were excluded, and the reasons for exclusion were documented. Any missing information relevant to this systematic review was requested from the corresponding authors via email.

Two reviewers independently extracted all relevant data from the included studies using a data extraction form specifically designed for this review.

The extraction sheet included the following information: study author, year of publication, trial registration, sample size (initial and final), age and sex of the participants, inclusion criteria, implant system, implant type and diameter, implant brand, number of implants placed, attachment design, prosthesis type, surgical procedure, success and survival rates, patient satisfaction, implant-related complications, follow-up length, and prosthetic failure.

The extracted data were systematically analyzed, and key information was presented in a standardized format.

Quantitative data were tabulated to facilitate risk ratio (RR) analysis with 95% confidence intervals (CIs).

The contributions (weights) of each study were included in the meta-analyses.

Dichotomous outcomes were analyzed using RRs with 95% confidence intervals (CIs), and continuous outcomes were analyzed using mean differences (MDs) with 95% CIs. Statistical significance was set at *P* < 0.05.

A random-effects model was used to account for variability between studies, and funnel plots were created to determine whether there was a possibility of publication bias.

### Assessment of risk of bias

Because this review involved comparative interventions, the methodological quality of the included RCTs was evaluated using the Cochrane Risk-of-Bias Assessment Tool. Two investigators independently assessed the methodological quality of the included studies.

### Statistical methods

All statistical analyses were conducted using RevMan version 5.4.1. The meta-analysis evaluated the MDs between the groups with the corresponding 95% CIs. Statistical significance was set at *P* < 0.05. Heterogeneity among the studies was assessed using the chi-square test and I² statistics. A random-effects model was applied to all meta-analyses conducted in this review.

## Results

### Search results

**Fig. 1:**
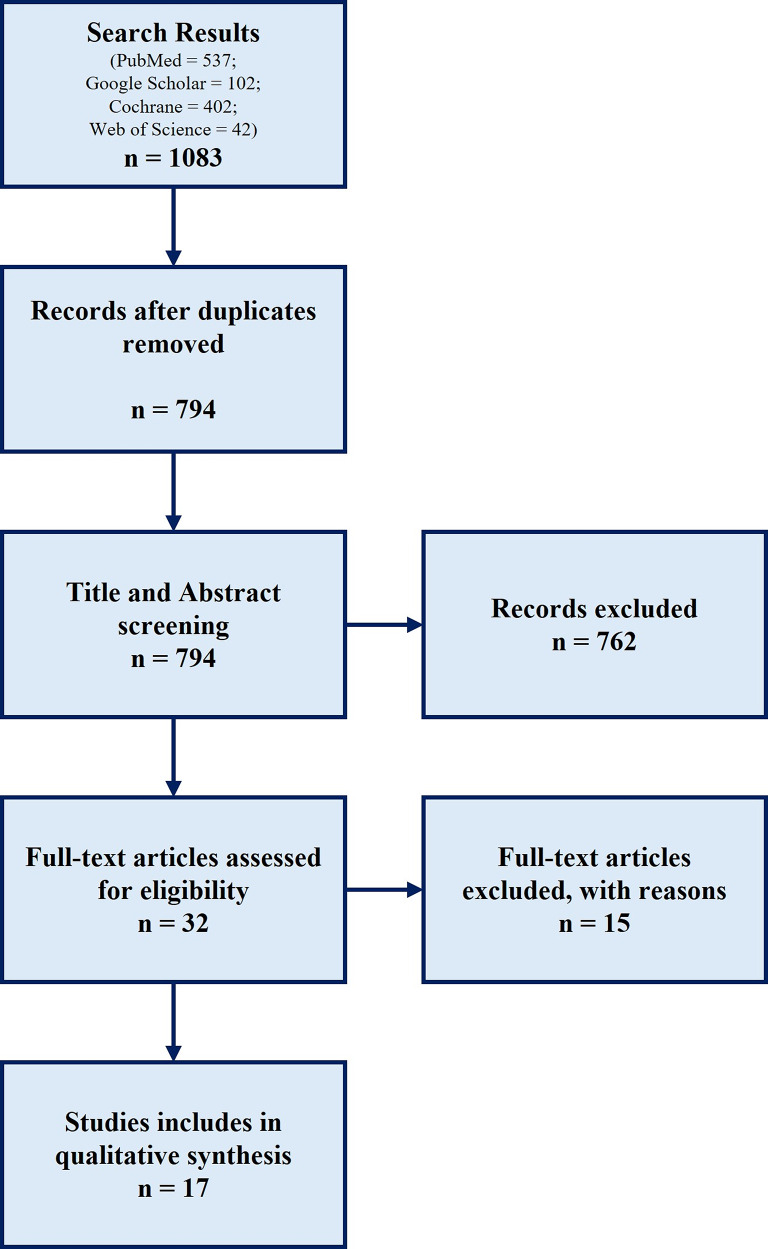
Flowchart of the review process

A total of 1,083 articles were identified. After excluding 289 duplicates, 794 articles remained. After screening the titles and abstracts, 762 articles were excluded, leaving 32 articles for full-text review. Of these, 15 articles were excluded: two for unavailable full text, six for duplicate data, six for an observation period of less than 1 year, and one for insufficient data. Finally, 17 [14–30] articles were included in the final analysis (Fig. 1). The excluded articles and the reasons for exclusion are summarized in Table 2, while the included studies and their summaries are presented in Table 3.

**Table 2:** Summary of excluded studies and corresponding reasons

Authors	Year	Reason for exclusion
Alfallaj HA	2018	Not enough results
de Resende GP et al.	2023	Data repeated in another included article
de Resende GP et al.	2021	Follow-up < 12 months
de Resende GP et al.	2021	Data repeated in another included article
Hartmann R et al.	2020	Data repeated in another included article
Hauck KE et al.	2021	Follow-up < 12 months
Heba Wageh Abozaed	2023	Full text not available
Jayasinghe RM et al.	2024	Follow-up < 12 months
Kant B et al.	2019	Follow-up < 12 months
Kronstrom M et al.	2014	Data repeated in another included article
Kronstrom M et al.	2010	Data repeated in another included article
Ranganathan DS	2020	Full text not available
Ravishankar DR et al.	2020	Follow-up < 12 months
Sousa CG et al.	2023	Follow-up < 12 months
Walton JN et al.	2010	Data repeated in another included article

**Table 3:** Summary of included studies

Study ID	Follow-up (months)	Patient age (years)	Loading time	Type of attachment	Implant system	Implant dimensions (mm)
**Al-Gazzar AE 2024** [13]	12	50–65 (range)	3 M	Ball	Is-II active, Neobiotech Implant System	3.5 × 10
**Alkhodary MA 2024** [15]	60	50–60 (range)	3 M	Ball	Sterngold PUR NP implants	3.2 × 14
**Alqutaibi AY 2017** [16]	12	58.2 (mean)	3 M	Locator	Dentis Implant System	NR
**AlSourori AA 2018** [12]	12	1-IOD 59 (mean) 2-IOD 57.4 (mean)	3 M	Locator	Dentis Implant System	3.7 × 10
**AlSourori AA 2023 **[28]	36	56 (mean)	3 M	Locator	Dentis Implant System	3.7 × 12
**Bryant SR 2015** [17]	60	67 (mean)	6 W	Ball	Solid Screw, SLA surface; Straumann	Length: 10 and 12
**Dewan H 2023** [18]	12	64.5 ± 2.3 (mean)	Immediate	Locator	Touareg-OS, ADIN Dental Implant System	NR
**Fahd AA 2018** [14]	12	58.2 (mean)	3 M	Locator	NR	NR
**Hartmann R 2020 **[19]	12	63.5 ± 8.8 (mean)	3 M	Ball	Titamax TI Cortical, Neodent	Diameter: 3.75
**Kronstrom M 2017 **[20]	60	53.3 (mean)	Immediate	Ball	Brånemark TiUnite	Length: at least 10
**Leles CR 2024 **[21]	60	65.4 ± 8.5 (mean)	21 D	Ball	SLActive, Straumann	4.1 × 8, 10, 12
**Paleari AG 2017** [22]	12	65.0 ± 10.2 (mean)	༔M	Ball	Conexão Sistema de Prótese Ltda; Arujá	3.75 × 11.5
**Patil PG 2020** [23]	12	64 (mean)	Immediate	Locator	Roxolid SLActive, Straumann	3.3, 4.1 × 10, 12
**Patil PG 2021** [24]	12	63.5 (mean)	Immediate	Locator	Roxolid SLActive, Straumann	3.3, 4.1 × 10, 12
**Policastro VB 2019** [25]	12	1-IOD 64.4 ± 8.3 (mean) 2-IOD 64.0 ± 6.4 (mean)	༔M	Ball	Conexão Sistema de Prótese Ltda, Arujá	3.75 × 11.5
**Tavakolizadeh S 2015** [26]	12	59 (mean)	Immediate	Ball	Simple line II, SOFX483810R, Implantium	Diameter: 3.8
**Yadav N 2022** [27]	12	57.38 ± 6.35 (mean)	2 W	Ball	Adin Dental Implant System	3.5 × 11.5

Studies in which no events occurred in either group were excluded from the meta-analysis because the RR could not be calculated for these comparisons.

### Implant failure (1Y/3Y/5Y)

**Fig. 2:**
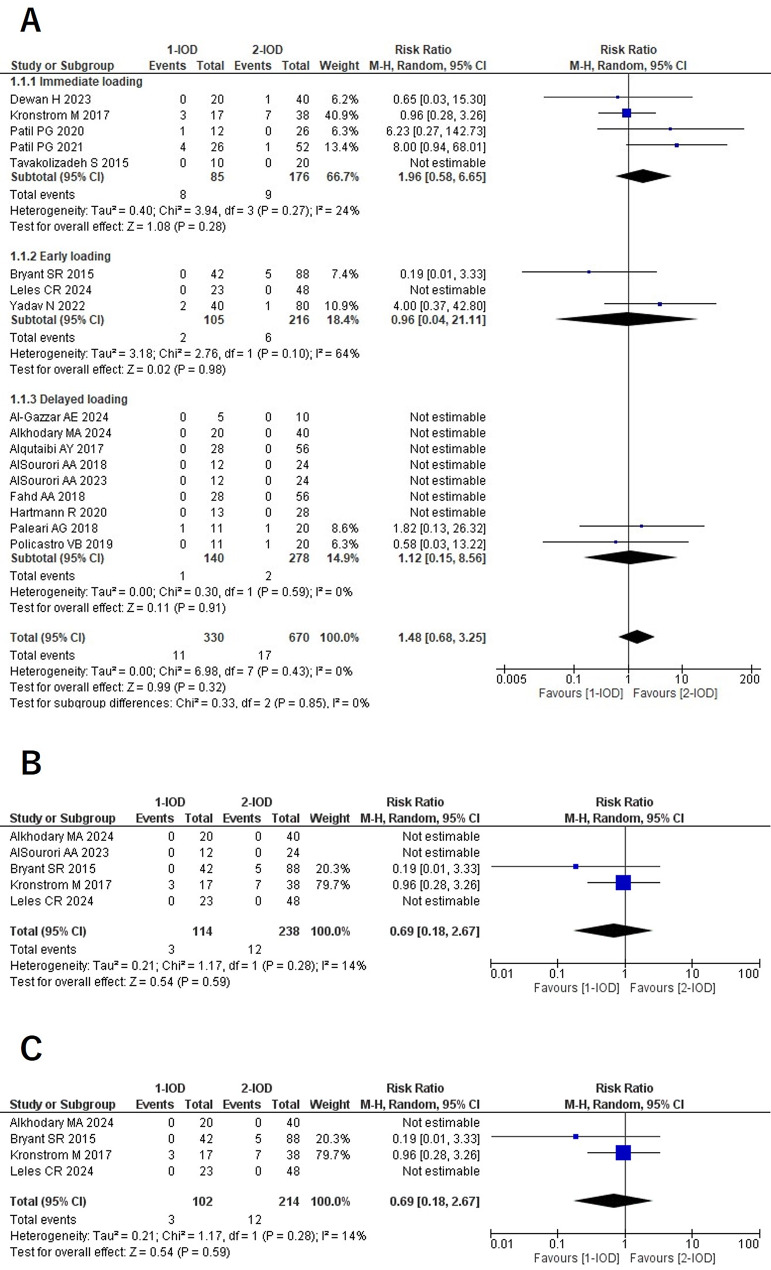
Forest plots of implant failure at the 1-year (**A**), 3-year (**B**), and 5-year (**C**) follow-up

No statistically significant differences in implant survival rates were observed between the 1- and 2-IOD groups at the 1-, 3-, and 5-year follow-ups. In addition, subgroup analyses based on loading protocols revealed no significant differences between the groups (Fig. 2).

### Denture fracture (1Y/3Y/5Y)

**Fig. 3:**
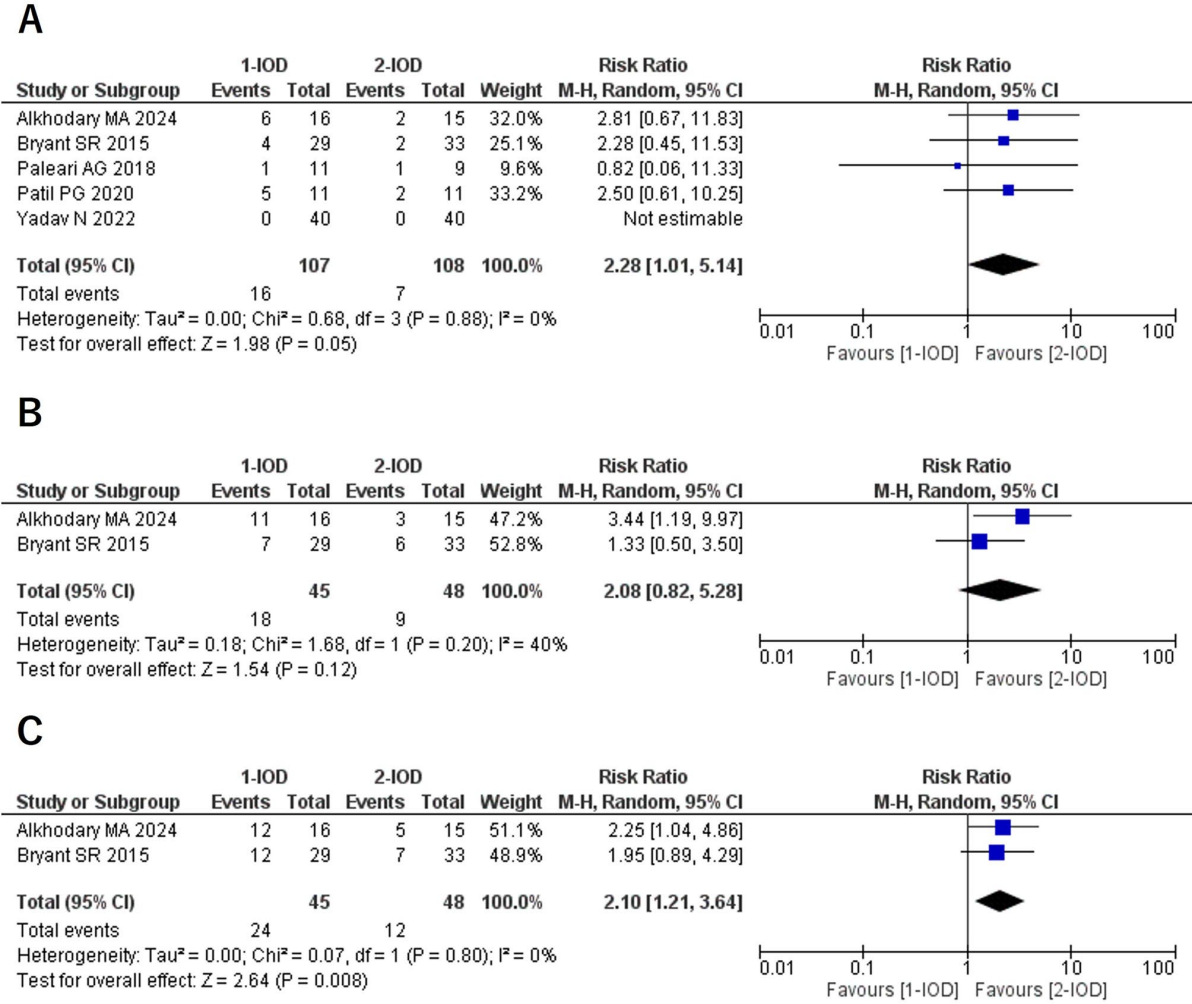
Forest plots of denture fracture at the 1-year (**A**), 3-year (**B**), and 5-year (**C**) follow-up

Regarding denture fractures, a statistically significant difference was observed at 1 year, with an RR of 2.28 (95% CI: 1.01–5.14, *P* = 0.05, I² = 0%, P for heterogeneity = 0.88). At 3 years, no statistically significant difference was found, with an RR of 2.08 (95% CI: 0.82–5.28, *P* = 0.12, I² = 40%, P for heterogeneity = 0.20), although mild heterogeneity was present. At 5 years, the RR was 2.10 (95% CI: 1.21–3.64, *P* = 0.008, I² = 0%, P for heterogeneity = 0.80), indicating that the risk of denture fracture in the 1-IOD group was approximately twice as high as in the 2-IOD group (Fig. 3).

### New denture fabrication (3Y/5Y)

**Fig. 4:**
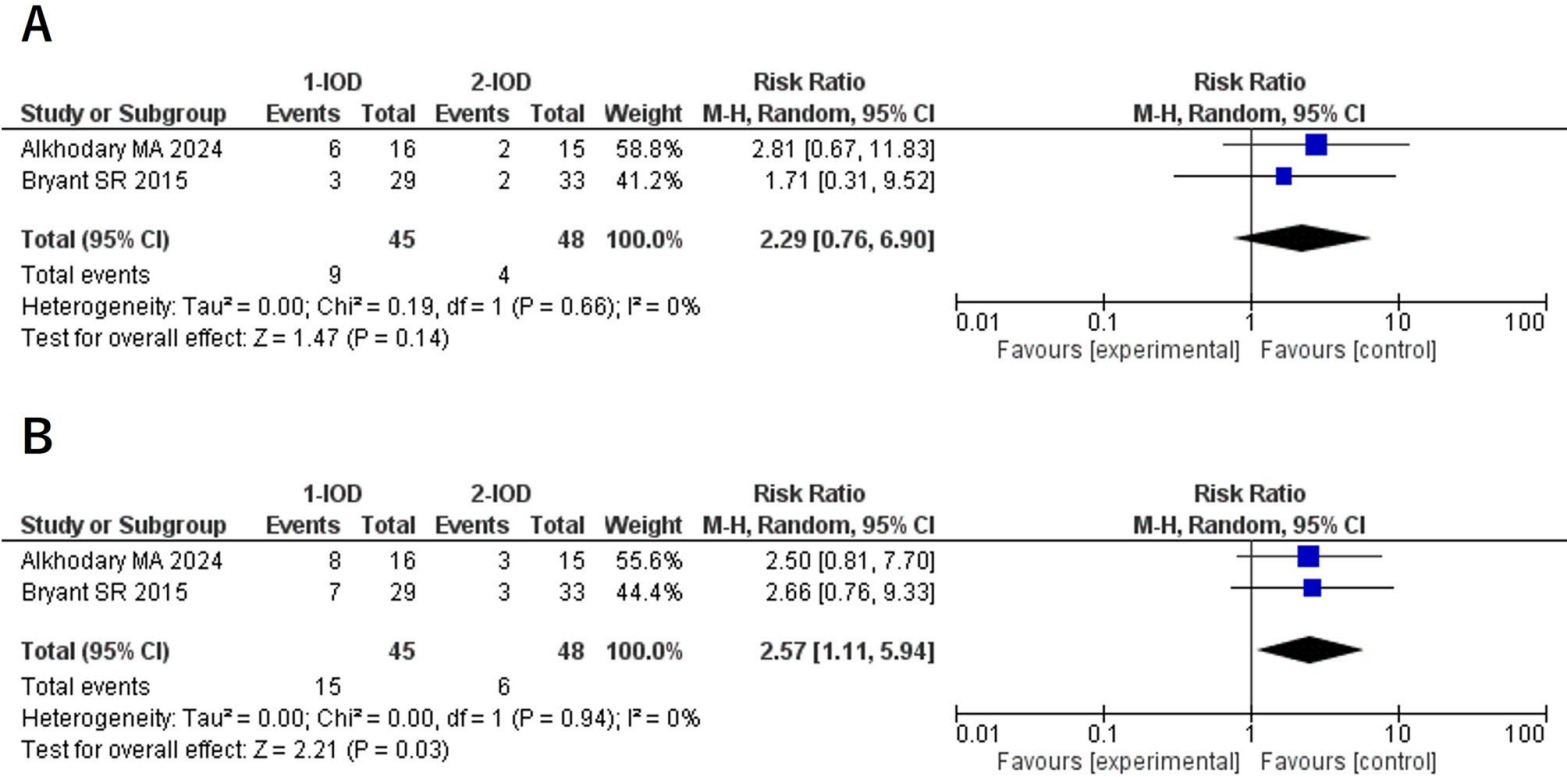
Forest plots of new denture fabrication at the 3-year (**A**) and 5-year (**B**) follow-up

No cases of denture replacement within 1 year were reported in any of the included studies; therefore, no meta-analysis was conducted for this period. At 3 years, the RR was 2.29 (95% CI: 0.76–6.90, *P* = 0.14, I² = 0%, P for heterogeneity = 0.19), and no statistically significant difference was observed. However, at 5 years, the RR was 2.57 (95% CI: 1.11–5.94, *P* = 0.03, I² = 0%, P for heterogeneity = 0.94), indicating that denture replacement was more common in the 1-IOD group than in the 2-IOD group (Fig. 4).

### Relining (1Y/3Y/5Y)

**Fig. 5:**
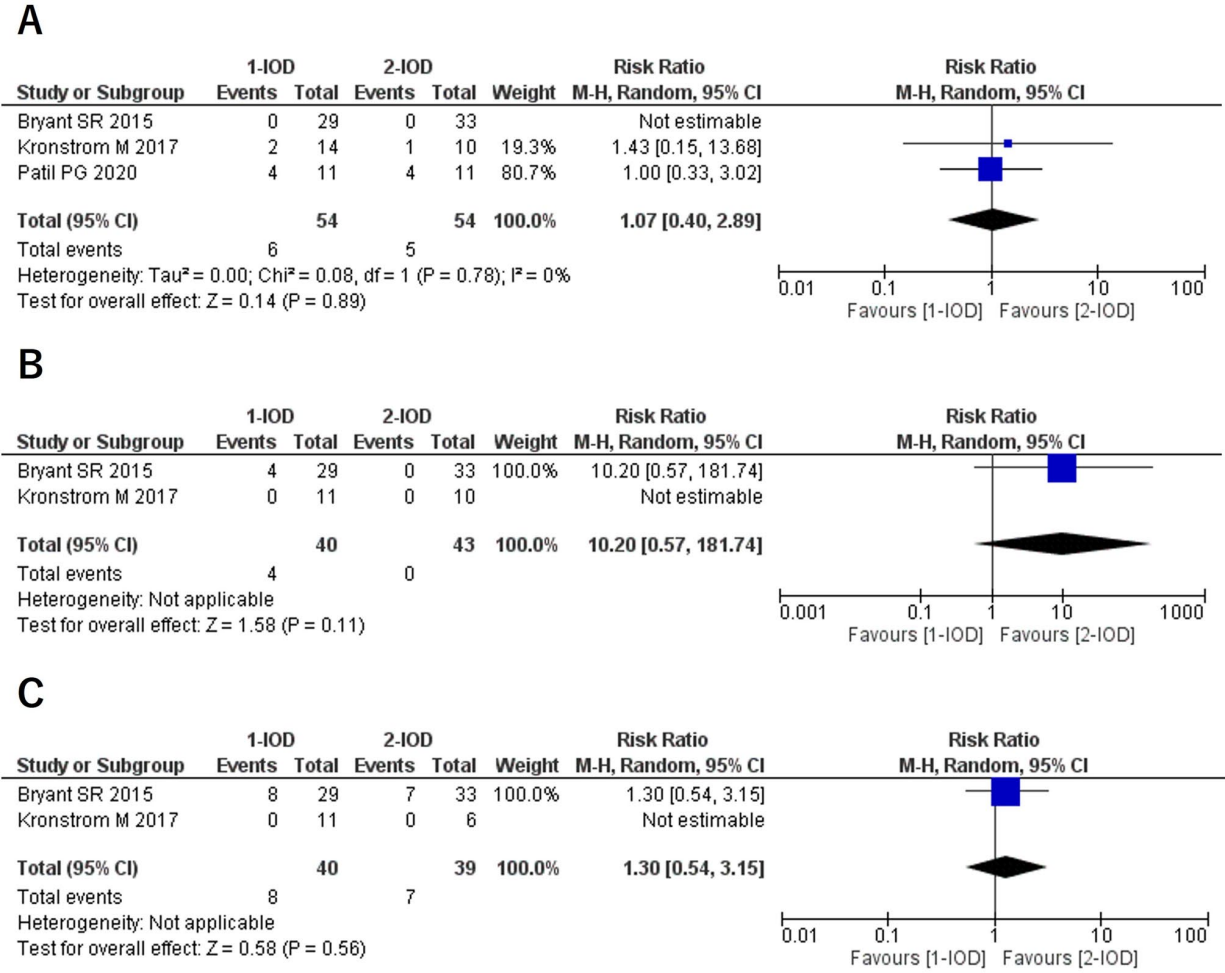
Forest plots of relining of mandibular overdenture at the 1-year (**A**), 3-year (**B**), and 5-year (**C**) follow-up

No statistically significant differences were observed in the frequency of mandibular IOD relining at 1, 3, and 5 years of follow-up (Fig. 5).

### O-ring replacement (1Y/3Y/5Y)

**Fig. 6:**
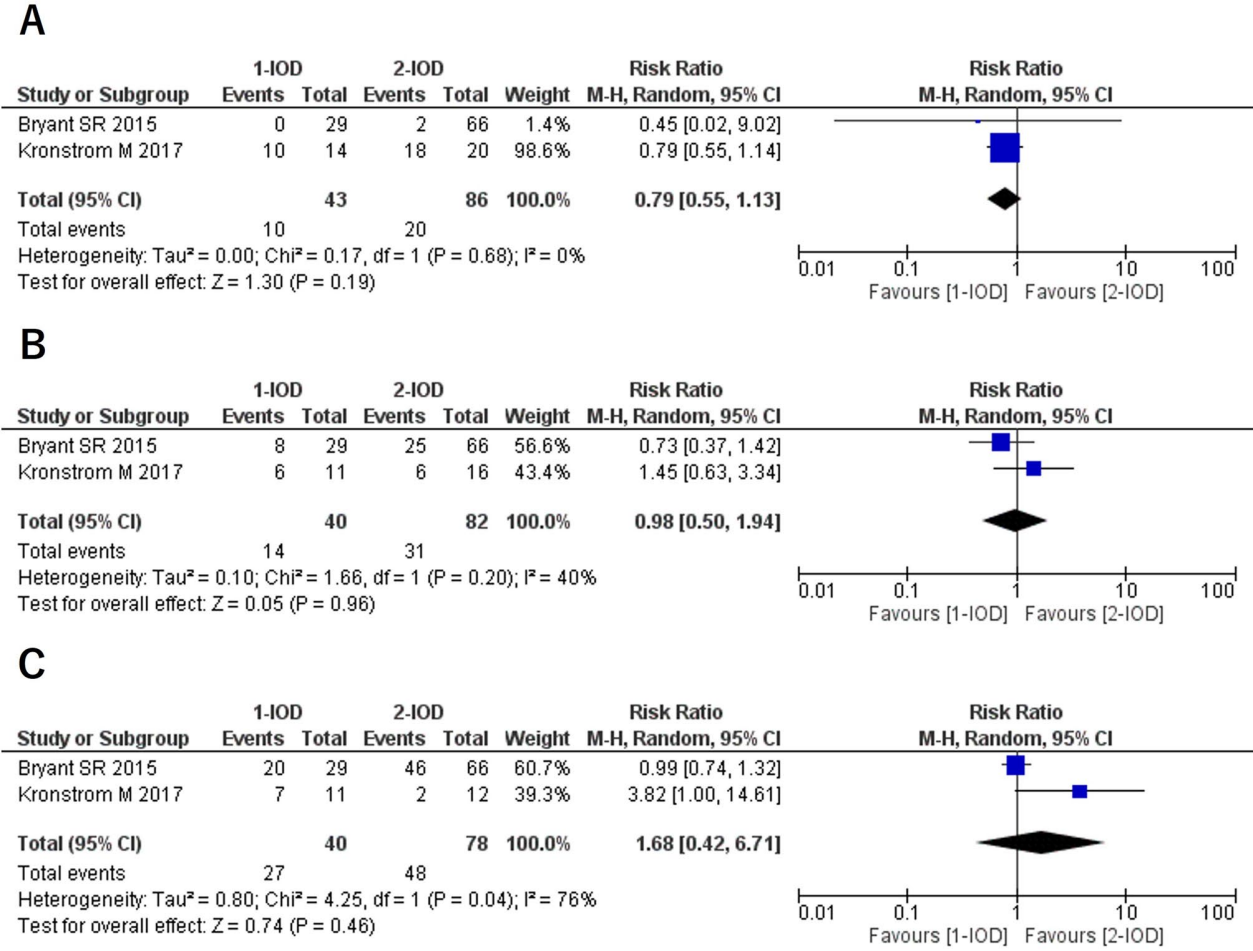
Forest plots of O-ring replacement at the 1-year (**A**), 3-year (**B**), and 5-year (**C**) follow-up

No statistically significant differences were observed in the frequency of O-ring replacement at 1, 3, and 5 years of follow-up (Fig. 6).

### Reattachment of metal housing (1Y/3Y/5Y)

**Fig. 7:**
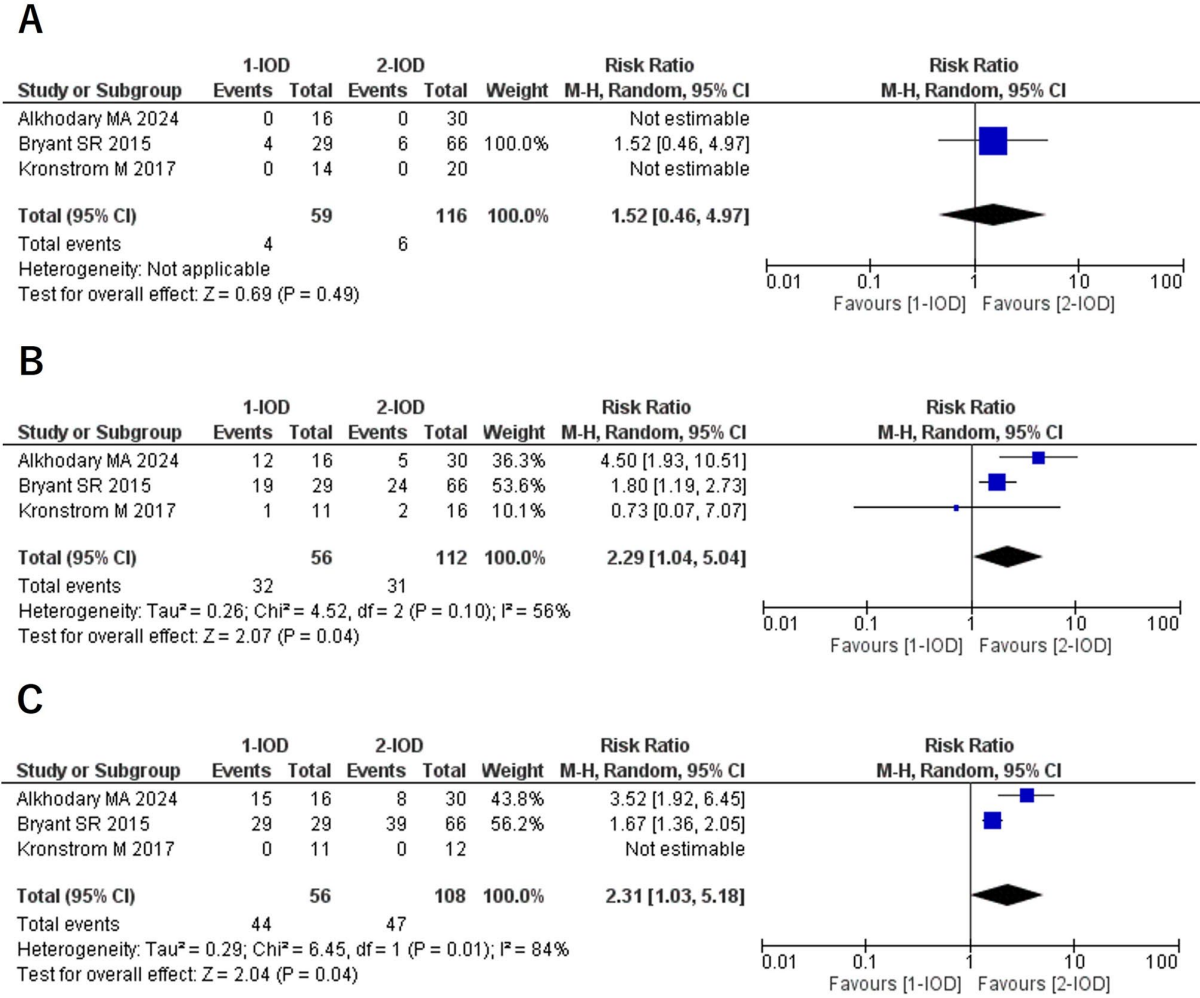
Forest plots of reattachment of metal housings at the 1-year (**A**), 3-year (**B**), and 5-year (**C**) follow-up

No statistically significant difference was observed in the frequency of metal housing reattachment at 1-year follow-up. However, at 3 years, the RR was 2.29 (95% CI: 1.04–5.04, *P* = 0.04, I² = 56%, P for heterogeneity = 0.10), and at 5 years, it was 2.31 (95% CI: 1.03–5.18, *P* = 0.04, I² = 84%, P for heterogeneity = 0.01), both indicating statistically significant differences. However, substantial heterogeneity was noted in the 5-year follow-up comparison (Fig. 7).

### Risk of bias assessment

**Fig. 8:**
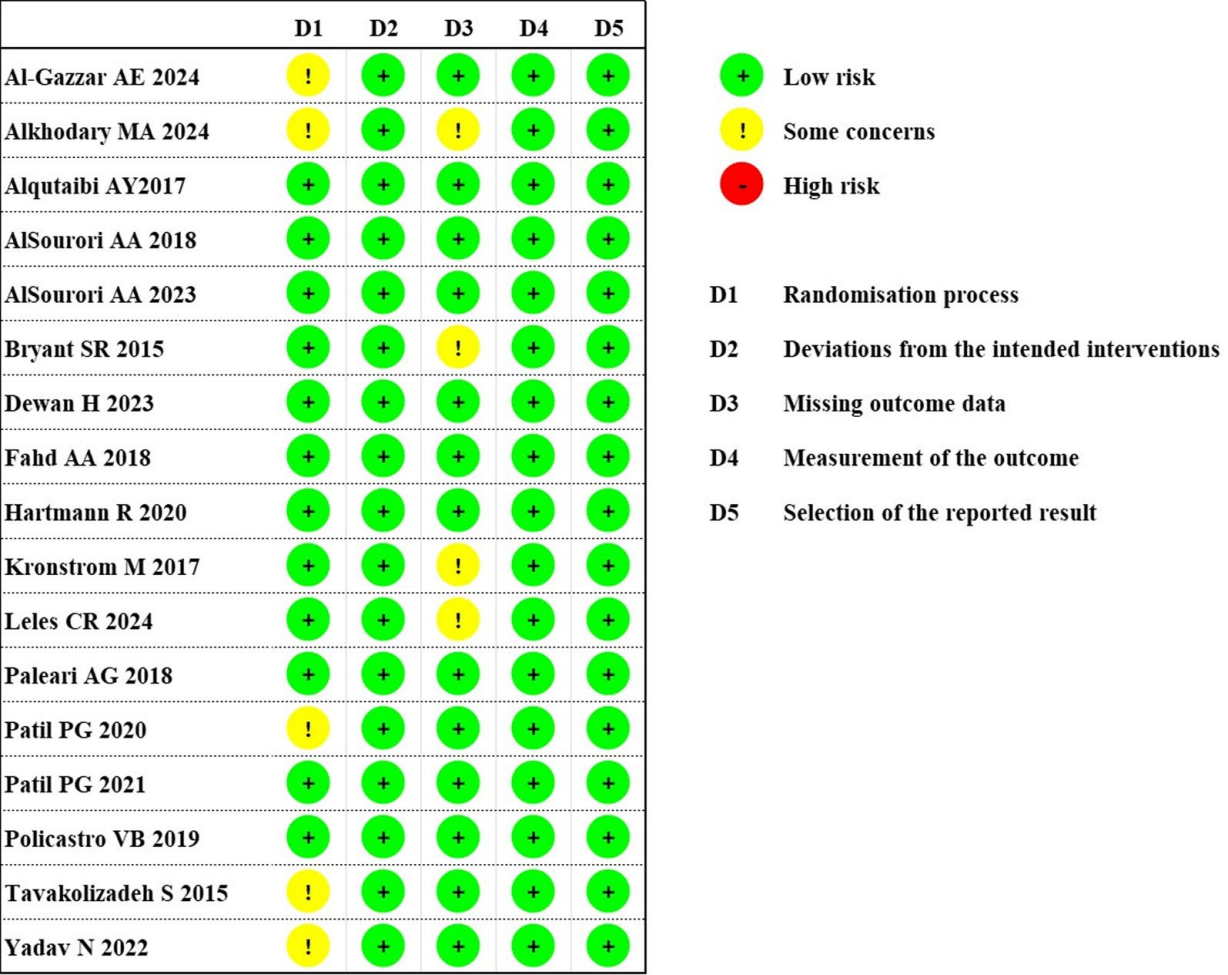
Risk of bias assessment for each included study using the Cochrane RoB 2 tool

The risk of bias in the included studies was assessed using the Cochrane Risk of Bias 2 (RoB 2) tool. Most studies were judged to have a low risk of bias across several domains. However, some studies did not provide sufficient information regarding the randomization process, resulting in a rating of “some concerns” in that domain. Furthermore, studies with a 5-year follow-up period exhibited a high proportion of missing outcome data, which also contributed to a rating of “some concerns” in the domain of missing outcome data (Fig. 8).

### Publication bias assessment

**Fig. 9:**
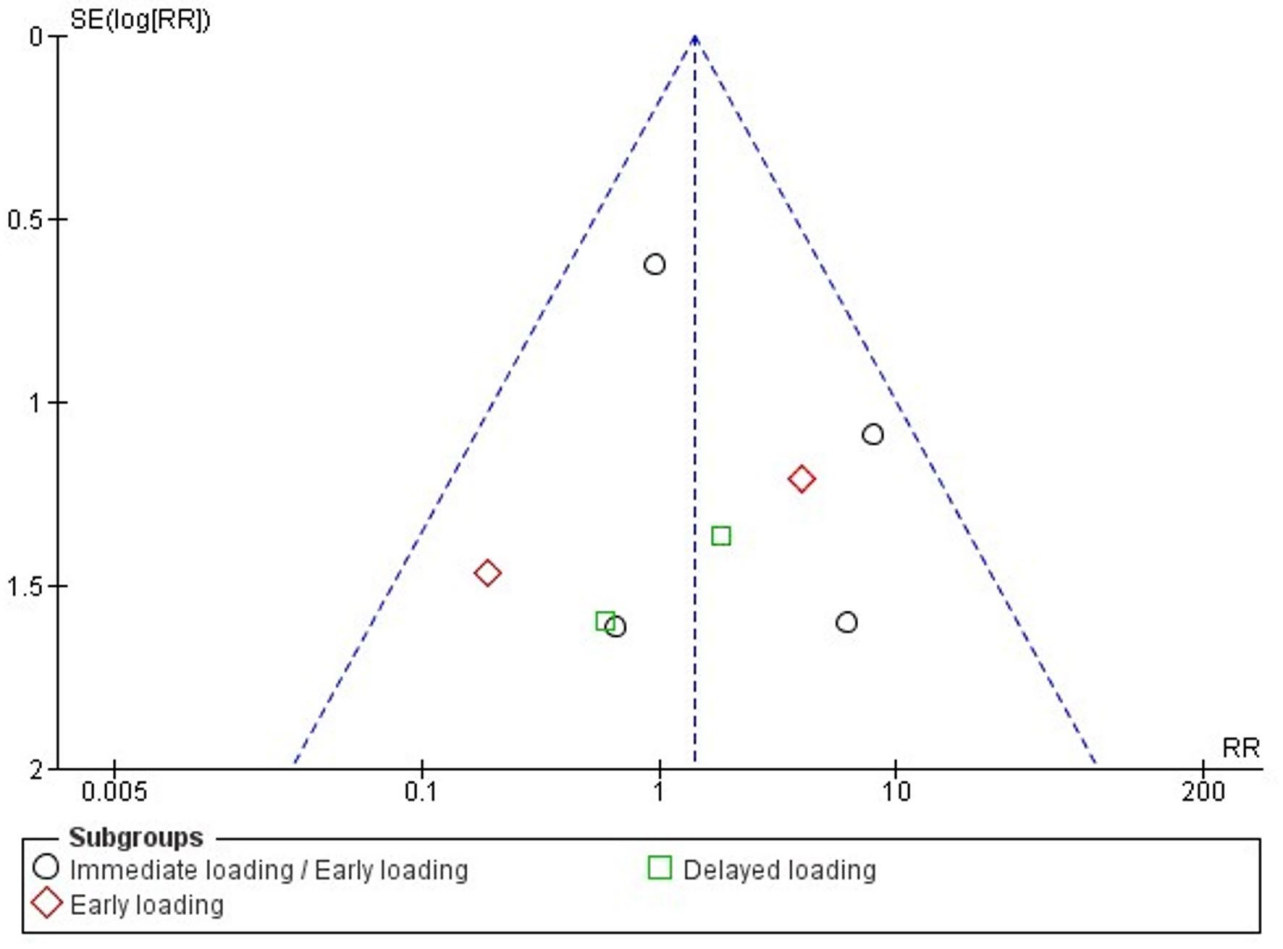
Funnel plots of implant failure at the 1-year follow-up

In the assessment of publication bias, the funnel plot showed a symmetrical funnel shape, indicating a low risk of publication bias and substantiating the validity of the meta-analysis (Fig. 9).

## Discussion

In recent years, the average global life expectancy has continued to increase. According to projections by the World Health Organization, by 2030, one in six people worldwide will be aged ≥ 60 years. Furthermore, the number of people aged ≥ 60 is expected to rise from approximately 1 billion in 2020 to 2.1 billion by 2050, while the population aged ≥ 80 years is projected to triple, reaching approximately 426 million [31].

As the global population continues to age, the number of older adults experiencing tooth loss is also expected to grow, leading to an anticipated increase in the number of patients with edentulism [32, 33]. Consequently, the demand for implant overdentures, one of the treatment options for edentulism, may rise in the future. This underscores the need for further accumulation of clinical and scientific evidence regarding this treatment modality.

Considering these demographic trends, this systematic review significantly updated the existing evidence by incorporating the latest RCTs. We evaluated the implant survival rate and prosthetic complications of 1- and 2-IODs. Although no difference in implant survival was observed between the 1- and 2-IOD groups, longer follow-up periods indicated that 1-IOD was associated with higher rates of denture fractures, fabrication of new dentures, and reattachment of metal housings than 2-IOD. A previous systematic review by Ahmed et al. reported a higher implant survival rate for 1-IODs than for 2-IODs [34]. However, after including the most recent RCTs published since 2017, our updated analysis found no statistically significant difference in implant survival between the 1- and 2-IOD groups. Subgroup analyses based on loading protocols also revealed no significant differences between the groups. Several reports support immediate or early loading protocols for mandibular implant overdentures, and our results are consistent with these findings [35–37].

In addition, long-term prosthetic complications, such as denture fractures, the need for new denture fabrication, and reattachment of metal housings, were found to occur more frequently in 1-IOD. These findings partially differ from those of previous systematic reviews [38].

Denture fractures occurring around the attachment area during function or insertion are thought to be related to changes in the distribution of occlusal forces when an overdenture is placed in the oral cavity. The implant–abutment junction may function as a fulcrum during the rotational movement of the overdenture under load, potentially increasing the risk of fracture at this site. However, because no significant difference in the frequency of relining was observed between the 1- and 2-IOD groups, the increased fracture incidence associated with 1-IOD is more likely due to differences in structural strength rather than increased denture mobility. Therefore, the use of a metal framework should be considered to reinforce morphology-dependent mechanical strength.

El-Zawahry et al. conducted a finite element analysis using a model in which a 100 N vertical load was applied to the right first molar region to simulate the natural masticatory condition of a patient with full dentition, where the food bolus was placed on one side. They observed that the total deformation of the 1-IOD model was approximately 50% greater than that of the 2-IOD model [39]. Moreover, the denture movement patterns differed between the 1- and 2-IOD groups. In 1-IOD, rotational movement under occlusal force is not limited to a single axis, resulting in different stress distributions [40].

In addition, in the case of 1-IOD, the Von Mises stress within the overdenture was reported to be higher than that in 2-IOD, potentially leading to an increased risk of denture fractures [39]. These differences are thought to result from variations in the support function provided by the number of implants, as well as the distinct patterns of denture movement controlled by the implants. Further investigations are required to clarify these mechanisms.

In IOD, the mucosal area of the residual ridge exhibits greater displaceability under pressure than the implant site, resulting in stress concentration around the attachment area. In 2-IOD, denture movement is restricted along the line connecting the two implants, which helps reduce the rotation and distortion of the denture. In contrast, 1-IOD lacks such a restriction, which may contribute to a higher risk of denture fractures. However, because fractures of dentures without metal framework reinforcement occur predominantly around the implant site [41–43], reinforcing this area with a metal framework may reduce the risk of denture fractures and the need for denture remaking. Finite element analysis of 1-IOD with a metal framework showed that the insertion of the framework reduced tensile stress around the implant housing by 61.8% [44]. Therefore, the incorporation of a metal framework into 1-IOD is recommended for the long-term prevention of denture fractures.

Implant prices vary depending on the country and clinic; however, Hartmann et al. reported that the cost of 1-IOD is approximately 75% of that of 2-IOD [45]. Considering that the number of implants and prosthetic abutments required for 1-IOD is approximately half of that required for 2-IOD, the initial cost is generally expected to be lower for 1-IOD. However, from a long-term perspective, the increased frequency of prosthetic complications may result in higher maintenance costs. Furthermore, if reinforcement with a metal framework is necessary to prevent fractures in 1-IOD, the initial cost will increase, potentially narrowing the cost difference between 1- and 2-IODs. Although 1-IOD is clearly less invasive than 2-IOD, further investigations are needed to determine the total cost, including maintenance.

Although the lack of standardized methods for evaluating patient satisfaction makes performing a meta-analysis challenging, multiple studies have consistently reported that 1-IOD significantly improves patient satisfaction compared with conventional complete dentures [16, 19–22, 24, 26–29]. Several studies have found significantly better outcomes for 2-IOD in terms of oral health-related quality of life, oral health impact profile scores, and questionnaire-based assessments [16, 20, 22, 24, 27, 29], whereas others have reported no significant difference between 1- and 2-IODs, suggesting that 1-IOD may serve as a viable alternative to 2-IOD [19, 21, 24, 28]. In fact, mixing ability tests using two-colored gums showed significant improvements in masticatory performance for both the 1- and 2-IOD groups compared with the baseline CD group, with no significant difference between the 1- and 2-IOD groups. These results were consistent with the patient satisfaction outcomes [21, 46]. Additionally, in masticatory performance tests using almonds, both groups demonstrated improved function compared to the CD group, and the 2-IOD group exhibited greater improvement than the 1-IOD group [24]. These findings suggest that improvements in masticatory function may be key factors contributing to increased patient satisfaction.

One of the limitations of this study is that most of the RCTs comparing 1- and 2-IODs had follow-up periods of 5 years or less, leaving the long-term prognosis largely uncertain. Furthermore, reports on prosthetic complications remain limited, indicating the need for further investigation.

Moreover, owing to insufficient sample sizes, subgroup analyses based on implant length, diameter, and type could not be performed. Therefore, additional RCTs are warranted to improve the precision of future meta-analyses and strengthen the overall evidence.

When deciding between 1- and 2-IODs, several factors should be considered, including the required level of retention and degree of prosthetic rotational freedom, as well as psychological, social, and economic aspects. Considering the increased maintenance demands, 1-IOD may be more suitable for patients who can attend frequent follow-up visits and wish to reduce initial treatment costs. Similarly, it could be suitable for cases in which 2-IOD placement is difficult owing to significant alveolar ridge asymmetry resulting from severe bone resorption in the canine or lateral incisor regions. Conversely, 2-IOD may provide greater prosthetic stability for patients who require stronger retention and support or have difficulty attending regular maintenance visits.

## Conclusions

This systematic review suggests that 1-IOD may serve as an alternative to 2-IOD. However, 1-IOD may be associated with a higher incidence of prosthetic complications, such as denture fractures and the need for new denture fabrication, than 2-IOD. These findings contribute to ongoing discussions in this field and provide evidence-based guidance to assist clinicians in making informed decisions to achieve optimal occlusal rehabilitation using implant overdentures tailored to individual patient circumstances. Nevertheless, as long-term observational data remain limited, further studies directly comparing 1- and 2-IOD, particularly focusing on the long-term incidence of prosthetic complications, are warranted to strengthen the current evidence.

## Data Availability

No datasets were generated or analysed during the current study.

## Abbreviations

1-IOD: Single implant-supported mandibular overdenture
2-IOD: Two implant-supported mandibular overdenture
CD: Complete denture
CI: Confidence interval
IOD: Implant-supported overdenture
MD: Mean difference
PICOT: Population, intervention, comparison, outcome, time
RCT: Randomized controlled trial
RoB 2: Risk of bias 2 tool
RR: Risk ratio
